# Methylation patterns associated with TTV load in geriatric hospitalized patients: an exploratory functional analysis

**DOI:** 10.1007/s11357-025-01938-6

**Published:** 2025-10-22

**Authors:** Carlo Fortunato, Pietro Giorgio Spezia, Federica Novazzi, Gretta Veronica Badillo Pazmay, Laura Cianfruglia, Francesca Drago Ferrante, Davide Gentilini, Luciano Calzari, Paolo Antonio Grossi, Nicasio Mancini, Luigi Rosa, Mirko Di Rosa, Anna Rita Bonfigli, Roberta Galeazzi, Antonio Cherubini, Leonardo Biscetti, Riccardo Sarzani, Fabrizia Lattanzio, Maurizio Cardelli, Fabiola Olivieri, Marco Malavolta, Francesco Piacenza, Fabrizio Maggi, Robertina Giacconi

**Affiliations:** 1 Advanced Technology Center for Aging Research, IRCCS INRCA, Ancona, Italy; 2 Laboratory of Virology, National Institute for Infectious Diseases, Lazzaro Spallanzani- IRCCS, Rome, Italy; 3 Department of Medicine and Technological Innovation, University of Insubria, Varese, Italy; 4 Laboratory of Microbiology, ASST Sette Laghi, Varese, Italy; 5 Department of Brain and Behavioral Sciences, University of Pavia, Pavia, Italy; 6 Bioinformatics and Statistical Genomics Unit, Istituto Auxologico Italiano IRCCS, Cusano Milanino, Milan, Italy; 7 Infectious and Tropical Diseases Unit, ASST-Sette Laghi, Varese, Italy; 8 Unit of Geriatric Pharmacoepidemiology and Biostatistics, IRCCS INRCA, Ancona, 60124 Italy; 9 Scientific Direction, IRCCS INRCA, Ancona, Italy; 10 Clinic of Laboratory and Precision Medicine, IRCCS INRCA, Ancona, 60121 Italy; 11 Geriatria, Accettazione Geriatrica e Centro Di Ricerca Per L’invecchiamento,IRCCS INRCA, Ancona, 60127 Italy; 12 Department of Clinical and Molecular Sciences, Università Politecnica Delle Marche, Ancona, Italy; 13 Neurology Unit, IRCCS INRCA, Ancona, Italy; 14 Department of Clinical and Molecular Sciences (DISCLIMO), Università Politecnica Delle Marche, Ancona, Italy; 15 Internal Medicine and Geriatrics, Hypertension Excellence Centre’ of the European Society of Hypertension, IRCCS INRCA, Ancona, Italy

**Keywords:** Torque Teno Virus, Aging, Differential methylation analysis, Gene set enrichment analysis, Immunity

## Abstract

Torque Teno Virus (TTV) is a widespread commensal virus within the human virome, characterized by a high prevalence in human population and an unclear pathogenic role. Over the past three decades, TTV has garnered increasing attention due to its ability to establish lifelong chronic viremia, which intriguingly fluctuates among individuals in relation to immune competence status, with a typical peak after an organ transplantation, followed by a plateau and a slow decrease. The regulatory mechanisms underlying TTV infection remain elusive, and factors influencing its interactions with the immune system have yet to be identified. To explore this complex interplay, we analyzed DNA methylation patterns associated with TTV load in older adult hospitalized patients (mean age: 83.15 ± 7.49) from the PROMOTERA cohort. In this study, we present for the first time the identification of differentially methylated probes (DMPs) correlated to TTV load in our cohort. The statistically significant DMPs were located in genes involved in immune regulation and lipid metabolism. To further characterize these findings, we performed an exploratory enrichment analysis by applying several *p*-value thresholds, which yielded multiple gene lists derived from the sets of significant probes. Genes associated with this epigenetic signature were found to enrich functional pathways related to immune activation, leukocyte differentiation, and cytokine production, while additional significantly enriched gene sets were involved in cell–cell adhesion and cell migration processes. Since our analysis followed an exploratory approach, these results should be interpreted as hypothesis-generating and warrant further investigation.

**Graphical Abstract:**

**Figure:**
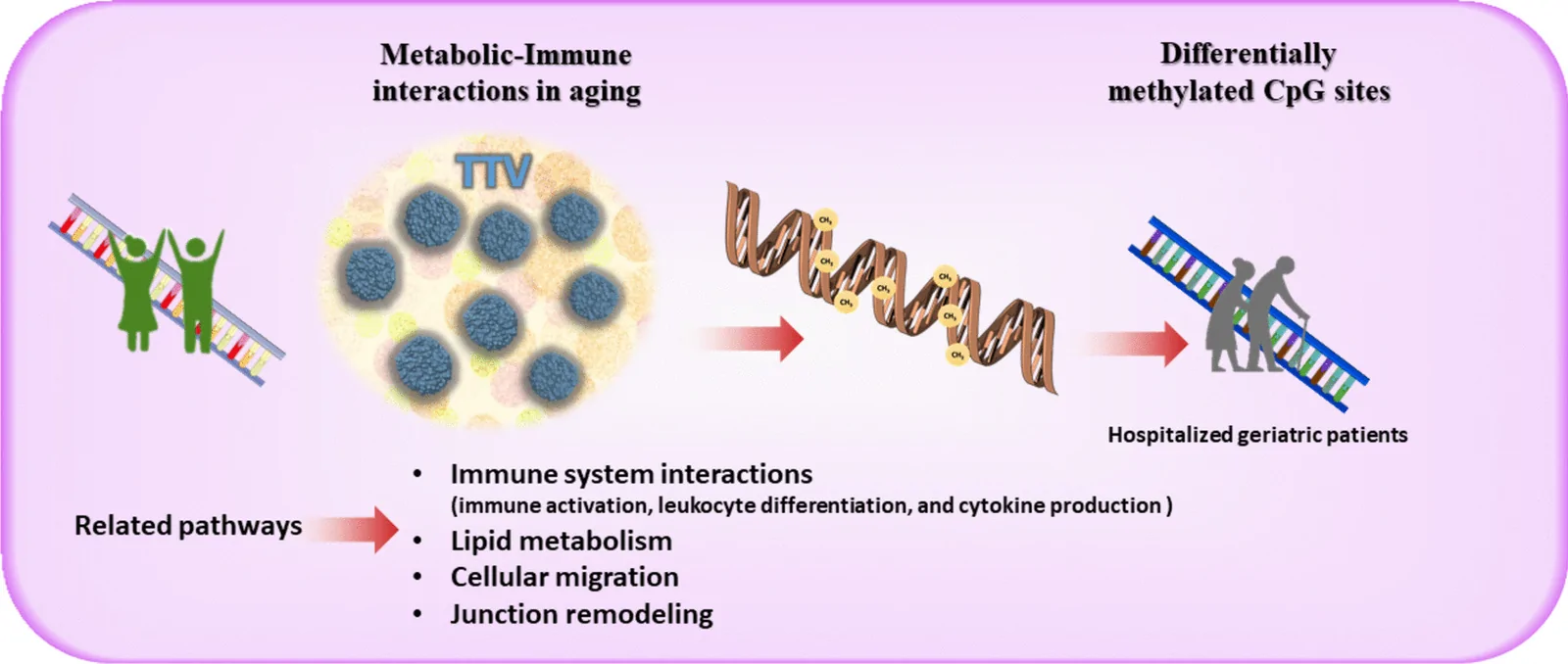

**Supplementary Information:**

The online version contains supplementary material available at https://doi.org/10.1007/s11357-025-01938-6.

## Introduction

TTV refers to a group of viral species belonging to the Anelloviridae family, which are the most representative members of the human virome [1]. The TTV genome consists of a circular negative single-stranded DNA of 3.8 kilobases located within a non-enveloped capsid with a diameter ranging from 30 to 50 nm [2]. Since its discovery, TTV load has been associated with various diseases affecting the liver, blood, immune, and respiratory systems [3]. Furthermore, it has been identified as a strong predictor of mortality in older adult people [4]. Nevertheless, the specific mechanisms by which TTV affects human pathology are mostly unclear. TTV is characterized by an extremely high prevalence in the worldwide population, which complicates the identification of a direct causative role in disease onset. Although a clear correlation with specific pathogenicity has not been established, growing evidence suggests a deep interplay between TTV species and immunomodulation: the viral replication has been demonstrated to occur in T lymphocytes in vivo and the viral load is inversely correlated with CD8^+^ 57^+^ T lymphocytes [5] and CD4^+^ T-cells [6]. The TTV genome encodes proteins with a high IgG reactivity [7], and TTV DNA can activate the TLR-9, leading to the production of pro-inflammatory cytokines [8]. In 2007, Zheng and colleagues observed that TTV ORF2 protein can downregulate the NF-κB (nuclear factor kappa-light-chain-enhancer of activated B cells) pathway, resulting in impaired transcription of IL-6, IL-8, and COX-2 [9]. Finally, the risk of rejection after solid organ transplantation is associated with low viral load, regardless of the type of organ [10, 11]. For these reasons, current research is focusing on investigating TTV as a marker of immune status.

With the advent of Next Generation Sequencing (NGS) technologies, large-scale data arrays can be collected from entire cohorts of subjects, enabling deeper insights into specific characteristics of the studied population, such as genetic variations, epigenetic modifications and transcriptomic profiles, thereby facilitating a more comprehensive understanding of healthy and disease mechanisms. DNA methylation is one of the most informative features in a broad range of biological processes, including tissue differentiation, aging mechanisms, behavioural aspects and pathologies [12, 13]. Due to its extensive applicability, variations in the methylome are the most studied epigenetic mechanisms, and numerous papers have been published investigating the relationship between methylation-driven regulation of gene expression and viral infections [14–17]. Several chronic viral infections have been associated with accelerated biological aging. For example, patients with chronic HBV or HCV infections, with or without HIV co-infection, show epigenetic age acceleration and correlations between DNA methylation patterns and immune cell markers [18]. Similarly, CMV infection has been linked to epigenetic remodeling in immune cells [19], while a targeted study has explored associations between CMV serostatus and methylation of aging-related loci such as ELOVL2 [20]. In contrast, despite its ubiquity and emerging clinical relevance, TTV has not yet been investigated in the context of DNA methylation changes, leaving a gap in our understanding of how persistent viral infections influence epigenetic regulation and immunosenescence in older adults.

Here we present a genome-wide methylation study conducted on an older adult hospitalized cohort in which we identified the epigenetic signature of TTV viremia. This study provides interesting insights into how the presence and load of TTV can influence specific methylation patterns that regulate gene expression. The variance of the differentially methylated positions (DMPs) detected in this study, can discriminate patients with low or high TTV loads. The functional annotation for the DMPs generated gene lists used for enrichment analysis [21], revealing significant enrichment in several immunity-related entries within the Gene Ontology functional database, highlighting the role of TTV in immune regulation and the epigenetic modulation of antiviral responses.

## Materials and methods

### Study population and data pre-processing

This study investigates the correlation between TTV viremia, measured as part of the VIROMA Project (grant no. PNRR-MAD-2022–12376334; ethics approval no. CE INRCA 23011, 12 April 2023), and DNA methylation status, assessed within the framework of the PROMOTERA Project (grant no. Grant Number GR-2019–12368606; ethics approval no. CE INRCA 20031, 04 February 2021). Both measurements were conducted on a cohort of 288 older adult patients (aged 62–102 years) selected from the PROMOTERA Project cohort [22], whose aim was to improve the prognostic value of multimorbidity (defined as the occurrence of at least two chronic conditions) through the integration of new methylation data to the already available clinical, functional, and biological routine data. In this cohort, DNA methylation levels at thousands of CpG sites were quantified in samples isolated from whole blood.

The cohort consists of older adults hospitalized at IRCCS INRCA between 2011 and 2019 as part of the Reportage Project [23]. This latter project aimed at creating a large data repository on demographics, comprehensive geriatric assessments, clinical and diagnostic information, and molecular and biological data on older adults receiving acute care.

Inclusion criteria for the PROMOTERA Project required participants to be over 65 years of age and presence of at least two chronic diseases, such as hypertension, dyslipidaemia, diabetes, acute or chronic ischemic heart disease, atrial fibrillation, cerebrovascular disease, stroke, chronic kidney disease, dementia, chronic obstructive pulmonary disease (COPD), osteoporosis, or thyroid dysfunction. Each of these diseases was classified as a binary variable referring to the absence or presence of the diagnosis.

### DNA extraction and Bisulfite conversion

DNA was extracted from whole blood using a commercially available kit (QIAmp® Blood Mini Kit). Each sample was mixed with lysis buffer at a 1:1 ratio and incubated with QIAGEN Protease at 56° for 15 min. Ethanol 96% (v/v) was then added to the mix at a 2:1 ratio. The mixture was loaded onto a QIAmp® Mini spin column and centrifuged at 20,000 × g for 2 min. After the washing steps, 70 µl of elution buffer was applied onto the dry column, and DNA was eluted through centrifugation at 6,000 × g for 3 min. Eluted DNA samples with aberrant 260/280 and 230/260 absorbance ratios were discarded or purified again. To detect the 5-methylcytosine residues, DNA was amplified and treated with bisulfite using the EpiTect® Fast DNA Bisulfite Kit, according to the manufacturer’s protocols.

### TTV load quantification

TTV loads in DNA extracted from blood samples were determined with RT-PCR as described in a previous study [24]. This assay employs primers designed on a highly conserved segment of the 5’-untranslated region (UTR) of the viral genome, thereby enabling the amplification of all TTV species.

### DNA methylation assay and data processing

Bisulfite-treated DNA samples were utilized for hybridization on on Infinium Methylation EPIC BeadChip850K, enabling the investigation of genome-wide methylated/unmethylated loci at a single nucleotide resolution. Fluorescence intensities were stored as intensity data files (*.idat) and processed using RStudio software (Version 2023.12.0.369 PBC, Boston, MA.). The R package “minfi” was used to calculate the methylation scores for each CpG site in the form of β-values or M-values [25]. Raw intensities of red and green channels were combined and preprocessed into a MethylSet. A RatioSet was obtained from the methylation and unmethylation channels and then normalized as performed in Genome Studio, using the preprocess Illumina() function implemented in “minfi”.

### Statistical analysis

Continuous variables were log-transformed when they did not exhibit a normal distribution, as assessed by the Shapiro–Wilk test. Group comparisons for continuous variables were performed using Student’s *t*-test or the Mann–Whitney *U* test, as appropriate, while categorical variables were compared using Pearson’s chi-squared test. Correlations were assessed using Pearson’s correlation coefficient. In multivariable analyses (e.g., differential methylation models), we adjusted for a set of relevant covariates to account for potential confounding factors. These included age, sex, hemoglobin levels (Hb), erythrocyte sedimentation rate (ESR), estimated blood cell-type proportions (CD4 + T cells, CD8 + T cells, and monocytes). All statistical analyses were performed using SPSS (Version 29.0.2.0 Armonk, NY: IBM Corp.) and RStudio software (Version 2023.12.0.369 PBC, Boston, MA.). Two-sided* p* values < 0.05 were considered statistically significant.

### Differential methylation analysis

The M-values matrix was filtered to exclude 8 CpGs exhibiting near-zero variance, and the final dataset included 866,230 CpGs. Linear regression models were fitted using the “limma” R package. Various design matrices were evaluated to optimize model sensitivity. The final model included log-transformed TTV viremia (logTTV), age, sex and the first two principal components representing the 74.08% of the explained variance of Hgb, Erytrocytes Sedimentation Rate (ESR), and the estimated proportions of CD8 T cells, CD4 T cells, and monocytes in samples (obtained from Steve Horvath’s Epigenetic Clock) [26]. Since our cohort exhibits numerous diseases, we performed an additional linear regression including the Charlson Comorbidity Index [27] into the design matrix to ensure that the episignature obtained was not influenced by the presence of pathologies.

All covariates were initially assessed for collinearity by calculating Pearson’s correlation coefficient. Nominal *p*-values were adjusted for multiple testing using the Benjamini–Hochberg method.

### Gene set enrichment analysis

Gene Set Enrichment Analysis (GSEA) was conducted using the WEB-based Gene Set Analysis Toolkit (WebGestAlt.org). To evaluate the enrichment of pathways associated with the episignature of TTV identified through DMA, four gene lists were generated by applying various *p*-value thresholds to the linear regression results. The GSEA utilized a list containing the gene symbols corresponding to each CpG site, which were annotated using the “IlluminaHumanMethylationEPICanno.ilm10b2.hg19” R package [28].

Gene lists were ranked by -log_10_(adj. *p*-value), logFoldChange (logFC) or logFC x -log_10_(adj. *p*-value) to capture both statistically significant and most influenced DMPs. Additionally, we analyzed by including only hypomethylated or hypermethylated probes, allowing us to observe gene sets emerging for a specific direction, referred to as the TTV effect on the methylome.

Enrichment analysis was performed with the number of permutations set to 1000 and an exponential phenotype scaling factor (p) of 1 for the enrichment score (ES) calculation. The sets analyzed were sourced from the “GeneOntology—Biological Process no Redundant” functional database [29], with the number of analytes in each category ranging from 5 to 2000.

Pathways with a false discovery rate (FDR) ≤ 0.05 and a *p*-value ≤ 0.05 were considered significant. However, sets with an FDR ≤ 0.25 were reported and considered for exploratory purposes.

Finally, we analyzed with the ClusterProfiler R package [30], setting the parameters as outlined for the WebGestAlt GSEA, to compare our method with a different enrichment algorithm.

### Principal component analysis

Since we aimed to observe how the methylation levels of the most correlated CpGs with TTV could effectively discriminate between patients with varying TTV loads, we applied dimensionality reduction techniques to the methylation data derived from the top episignature obtained through a linear regression model. We generated scatter plots showing the separation of patients with logTTV values below and above the median based on the first two components obtained, utilizing the “ggplot2” R package. To assess how the discriminatory capacity evolved along the ranked CpG signature, including those not reaching statistical significance after multiple testing correction, we performed an iterative PCA for each ranking score used in the GSEA. Starting from the top 5 CpGs, we progressively included larger subsets of the ranked episignature, extended the analysis to those probes with an adjusted *P*-value > 0.05, up to 1500 CpGs. For each PCA, we evaluated the distribution of patients grouped by logTTV (above and below the median) and quantified the separation between the two groups as the Euclidean distance between the centroids of each group in the PC1-PC2 space. Additionally, we trained logistic regression models using PC1 and PC2 as predictors to classify individuals according to their logTTV group, computing the area under the ROC curve (AUC) for each model using the “pROC” R package [31]. Finally, we recorded the cumulative variance explained by PC1 and PC2 at each iteration, measuring the dimensionality reduction effectiveness and noise accumulation.

### Cross-validation

Cross-validation models were implemented using the “glmnet” R package [32] to assess the predictive power of M-values across the entire methylome for logTTV. The models included (1) methylation data only, (2) methylation data, age and gender and (3) methylation data combined with all the covariates used in the differential methylation analysis. During the training phase, we determined an optimal alpha value of 1, corresponding to pure LASSO regression, which is particularly suitable for high-dimensional contexts and helps prevent data overfitting by imposing a stronger penalty on the selection of predictors. The number of folds was set to 10. The dataset was split into training and test sets in a 9:1 ratio to avoid data leakage. The resulting subsets were validated by comparing the distribution of logTTV.

For each model, the lambda value was selected to minimize the Mean Squared Error across all training iterations. Additional evaluations were performed to assess the normality of residual distributions. Finally, to validate the episignature derived from the "limma" regression model, we compared the top 50 CpGs of the logTTV signature with the predictors selected by each cross-validation model.

## Results

### Characteristics of the study cohort

The characteristics of the study population are summarized in Table 1, all patients included in the study were positive for TTV and were grouped by the median of TTV viremia (log-transformed). Lower values of logTTV were found in younger patients. Overall, the study cohort comprises very old subjects (mean age: 83.15 ± 7.49 years), including 131 males and 157 females, and shows a high prevalence of common chronic diseases such as hypertension (79.9%), diabetes (37.9%), chronic kidney disease (23.6%), and atrial fibrillation (19.1%).

**Table 1:** Characteristics of the study population

	**logTTV < median (*N*** **= 145)**	**logTTV ≥ median (*N*** **= 143)**	**Total (***N* **= 288)**	***P* value**
Age	82.54 ± 7.57	83.97 ± 7.27	83.15 ± 7.49	0.030
Diabetes n (%)	29 (20.00%)	30 (20.98%)	109 (37.9%)	0.837
Hypertension n(%)	90 (62.07%)	76 (53.15%)	230 (79.9%)	0.126
AF n (%)	24 (16.55%)	31 (21.68%)	55 (19.1%)	0.268
CHF n (%)	6 (4.14%)	4 (2.80%)	10 (3.5%)	0.750
CKD n (%)	30 (20.69%)	38 (26.57%)	68 (23.6%)	0.240
COPD n (%)	24 (16.55%)	19 (13.29%)	43 (14.9%)	0.437
Anaemia n (%)	21 (14.48%)	33 (23.08%)	54 (18.8%)	0.062
Malnutrition n (%)	6 (4.14%)	7 (4.90%)	13 (4.5%)	0.757
Dementia n (%)	18 (12.41%)	27 (18.88%)	48 (16.7%)	0.131
NLR	4.43 ± 4.24	4.73 ± 3.89	4.58 ± 4.07	0.299
Neutrophils (× 10^3^/µL)	5.40 ± 3.24	5.94 ± 3.53	5.66 ± 3.39	0.126
Lymphocytes (× 10^3^/µL)	1.53 ± 0.68	1.56 ± 0.75	1.54 ± 0.71	0.799
eGFR, mL/min/1.73 m^2^	58.34 ± 21.35	55.06 ± 19.95	56.68 ± 20.68	0.130
β-blockers	46 (31.72%)	49 (34.27%)	94 (32.6%)	0.646
Calcium channel blockers	22 (15.17%)	27 (18.88%)	49 (17.0%)	0.402
ACE Inhibitors	32 (22.07%)	27 (18.88%)	59 (20.5%)	0.503
Angiotensin II receptor antagonists	35 (24.14%)	33 (23.08%)	68 (23.6%)	0.832
Organic nitrates or other vasodilatators	7 (4.83%)	7 (4.90%)	14 (4.9%)	0.979
Statins	42 (28.97%)	47 (32.87%)	90 (31.3%)	0.474
Antithrombotic agents	86 (59.31%)	95 (66.43%)	183 (63.5%)	0.211
SIRI	2.56 ± 4.12	3.38 ± 8.30	2.97 ± 6.55	0.289

Data are shown as mean ± standard deviation. Groups comparison was conducted with Student’s *t*-test or Mann–Whitney *U* tests and with Pearson’s Χ^2^ test for continuous and binary variables, respectively

*AF* atrial fibrillation, *CHF* congestive heart failure, *CKD* chronic kidney disease, *COPD* Chronic obstructive pulmonary disease, *eGFR* estimated glomerular filtration rate, *NLR* Neutrophil-to-lymphocyte ratio, *SIRI* Systemic inflammation response index

When comparing patients for the viremia, age was significantly lower in individuals with TTV levels below the median (82.54 ± 7.57 vs 83.97 ± 7.27 years, *p* = 0.030). A higher prevalence of anaemia was observed in the group with higher TTV levels (23.08% vs 14.48%), with a trend toward statistical significance (*p* = 0.062). A comparison by gender (Table S1) revealed that female patients were significantly older than males (84.01 ± 7.13 vs 82.13 ± 7.81 years, *p* = 0.042). Chronic kidney disease was more prevalent among males, affecting 30.5% of men compared to 17.8% of women (*p* = 0.012). No other variables showed statistically significant differences.

### The TTV epigenetic signature

After collecting and processing the methylation data, we implemented a linear regression model using the “limma” R package to identify differentially methylated positions (DMPs) across the entire methylome in relation to the log-transformed TTV load (logTTV). To ensure robustness, we conducted several sensitivity tests by including different covariates in the design matrix. The resulting models were evaluated for their ability to identify significant correlations between logTTV and each CpG site (adjusted *P*-value < 0.05). However, the best model revealed only five significant sites (Table S2). This model included logTTV as a contrast for the fit, along with age, sex, ESR, Hgb and the first two principal components of cell type estimations in the samples, in particular monocytes, CD4^+^ T and CD8^+^ T lymphocytes. To filter out the methylome variance unrelated to logTTV, all continuous covariates were tested for collinearity using Pearson’s correlation method. Genomic annotation was subsequently retrieved from the “IlluminaHumanMethylationEPICanno.ilm10b4.hg19” R/Bioconductor package.

Notably, based on a PubMed search of the genes annotated to the DMPs, we found that the sites showing a significant correlation with viral load were mapped to genomic loci associated with the regulation of: i) immunity system-related disorders; ii) adipogenesis, adipocytes differentiation, or lipid metabolism in general. Table 2 provides the corresponding PubMed IDs, highlighting the involvement of these genes in immunity and/or lipid metabolism, respectively. Specifically, those loci significantly correlating with viral load included MROH9, which has been linked to the activation of innate immune signaling pathways [33–35]; ZNF638, involved in anti-viral immune response but also contributing in adipocyte differentiation [36, 37]; HOXB13, a homeobox gene involved in prostate cancer onset through epigenetic reprogramming of the histone deacetylase HDAC3. Notably, this protein regulates gene expression of lipogenic effectors [38–40]; the imprinted SGCE:PEG10 locus, which regulates cell proliferation and lipid homeostasis [41, 42]; and LPIN1, encoding a phosphatidate phosphatase critical for triglyceride biosynthesis and adipocyte maturation [43, 44].

**Table 2:** Literature-based functional annotation of top-ranking genes

Gene Symbol	Immunity	Lipid Metabolism
MROH9	35,077,391, 38,953,571, 39,919,905	–-
ZNF638	36,358,661, 39,464,150	21,602,272, 33,040,080
HOXB13	35,296,171, 38,341,319	35,468,964
SGCE:PEG10	32,976,381, 37,932,427, 16,225,771	–-
LPIN1	35,149,744, 35,222,395	35,149,744

PubMed IDs of current literature associating the top-ranking genes with immune functions or lipid metabolism

We assessed whether the inclusion of HOXB13 among the DMPs could have been biased by the presence of patients with a cancer diagnosis (13.9% of the cohort), of whom only 7 had prostate cancer. A separate analysis excluding these patients still identified HOXB13 among the significant DMPs (adjusted *P*-value < 0.04), suggesting that its differential methylation may reflect a broader biological implication, potentially linking viral load to lipid metabolism and inflammatory processes through epigenetic modulation. Notably, the episignature obtained from this test (Table S3) still showed the probes located in LPIN1, ZNF638, MROH9 and PEG10:SGCE among the top-ranking features, although with slightly reduced sensitivity. For this reason, we concluded the correlation identified with these probes and logTTV was independent of cancer diagnosis in our cohort.

Given the high prevalence of pathologies in the study population, we also investigated whether these comorbidities might affect the episignature obtained. For this reason, we performed an additional linear regression including the Charlson Comorbidity Index as a covariate in the design matrix and compared the results. The resulting episignature (Table S4) confirmed the same DMPs (adjusted *P*-value < 0.05) annotated to the genes mentioned above. To further support the robustness of our findings, we performed an overlap analysis including probes with a nominal *p*-value < 0.05, not significant after multiple testing correction. We found 81,652 probes in common out of 92,412 under this threshold (overlap 83.5%). We also calculated the correlation among the logFC values of the two models resulting in a Pearson’s coefficient of 0.9977, with a mean difference of 0.00015.

**Fig. 1:**
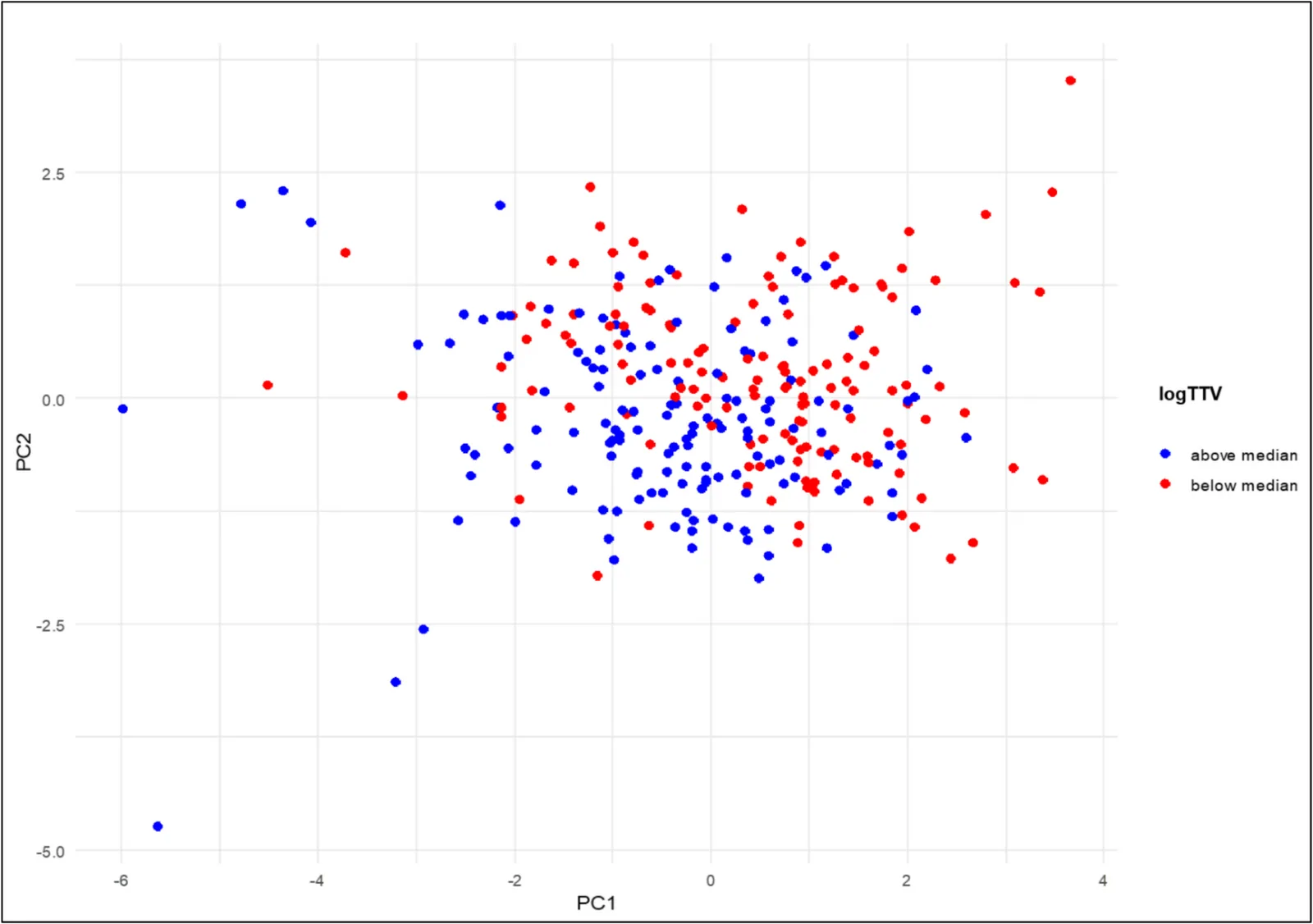
Principal component analysis of the CpGs M-values with adjusted *p* < 0.05. Patients are coloured according to their viral load, above (blue) or below median (red). Explained Variance: PC1: 0.34; PC2: 0.15; Pearson correlation: PC1/logTTV: ρ = −0.40; PC2/logTTV: ρ = −0.31

To further explore our findings, we first examined whether the methylation levels of the top CpG sites could differentiate patients according to their viral load. Figure 1 shows the scatter plot of patients, coloured according to logTTV values above and below the median, across the first two principal components derived from the M-values of the 5 significant sites.

**Fig. 2:**
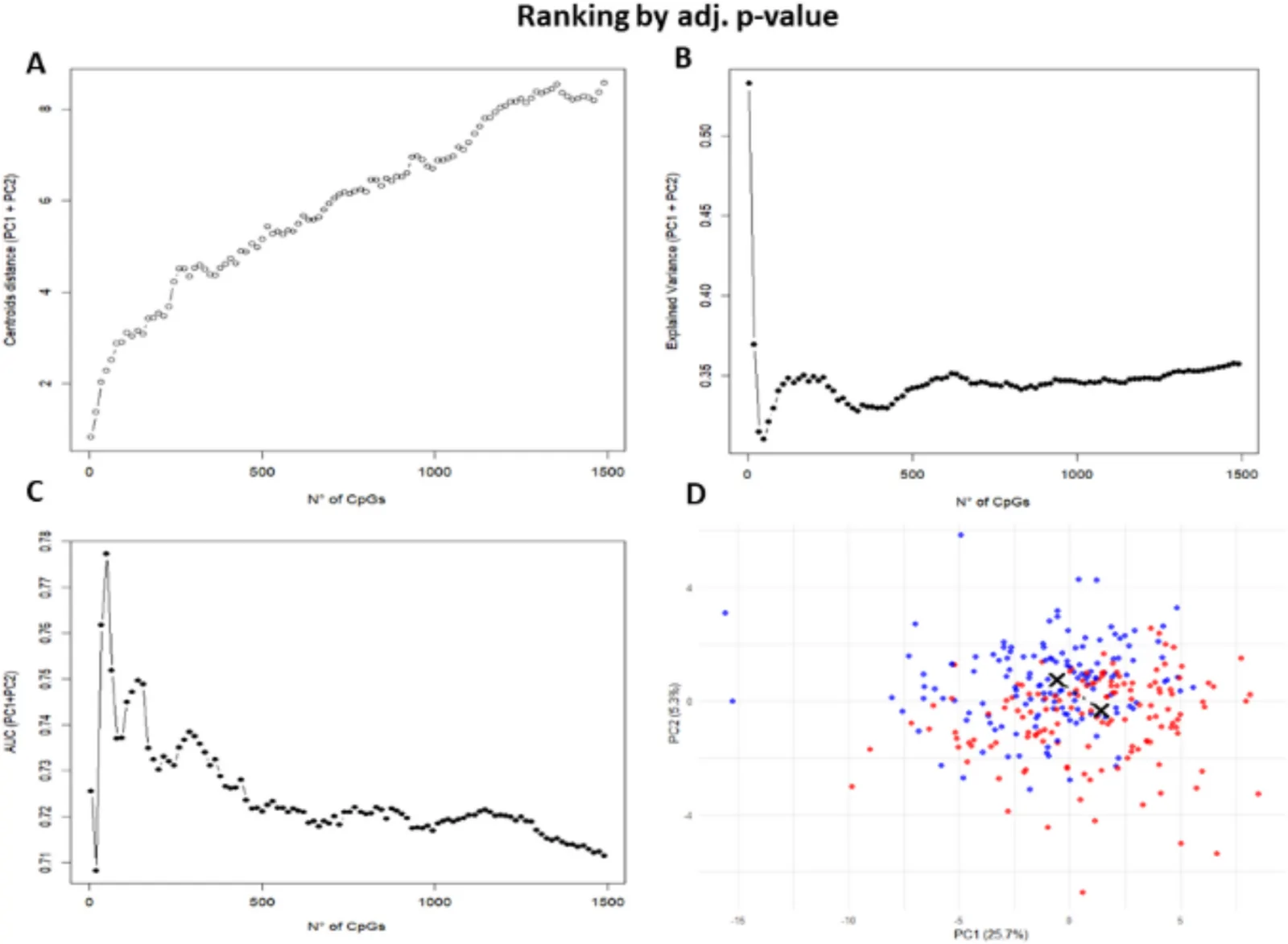
Iterative Principal Components Analysis for methylation levels ranked by adjusted *p*-value. (**A**) Euclidean distance of the centroids of the two clusters of patients (above and below median of logTTV) in the PC1 and PC2 space in function of the number of CpGs included in the PCA. (**B**) Variance explained by the first two components of the PCA in function of the number of CpGs included. (**C**) Area under ROC curve variation referred to the first two components predictivity towards the two groups of patients (above and below logTTV median) in function of the number of CpGs included in the PCA. (**D**) Scatter plot. Patients are coloured according to their viral load, above (blue) or below median (red). Centroids and their distance are shown as black crosses and a dotted line, respectively. Pearson correlation: PC1/logTTV: ρ = −0.36; PC2/logTTV: ρ = 0.55

Since the number of significant sites was insufficient for a functional enrichment analysis, we further examined whether the PCA could discriminate patients with logTTV values above and below the median by including the methylation levels of CpG sites with adjusted *P*-value > 0.05. Therefore, we performed an iterative PCA along the episignature ranked by adjusted *P*-value, evaluating how the first two components deriving from the M-values of the sites included allowed to visualize the separation between the two groups. For each iteration, we calculated the Euclidean distance between the centroids in the PC1-PC2 space (Fig. 2A) and the explained variance attributed to the first two components (Fig. 2B). Although the distance increases along with the number of probes included, the explained variance reaches the minimum at 50 sites (highlighted in blue in Table S2), indicating the first two principal components are no longer able to capture the discrimination and that, from this threshold, the methylation levels add noise to the analysis. To assess the predictive value of the first two components obtained from each iteration in distinguishing the two patient groups, we implemented a logistic regression model with the R package “pROC” and evaluated changes of the AUC. Consistent with our previous results, 50 sites resulted in the highest AUC value, corresponding to 0.721 (Fig. 2C). The corresponding scatter plot along the PC1 and PC2 obtained with these probes is reported in Fig. 2D. We then extended the iterative PCA approach to CpG site rankings based on alternative metrics used in the functional enrichment analysis and identified thresholds of probes needed in a PCA for the optimal separation of clusters of subjects above and below logTTV median. As shown in Fig. S1, the PC1 and PC2 of the methylation levels sorted by logFC obtained the highest AUC at 590 sites (AUC = 0.8871). Finally, considering the score logFC x -log10(adj. *p*-value), the best regression occurred at 365 sites (AUC = 0.859) (Fig. S2). We indeed observed an increased correlation between the principal components of the M-values of the top 50 sites and logTTV compared to the correlation obtained with the top sites (Fig. S1). Consequently, we decided to conduct an exploratory gene set enrichment analysis (GSEA), expanding the CpG list to include those probes with an adjusted *p*-value greater than 0.05. To assess the sensitivity of this approach, we applied different thresholds based on nominal *p*-values. As described in the Materials and Methods section, we tested four different lists (L1-L4) (Tables S3), varying the threshold of the probes’ nominal *p*-value and calculated several ranking scores for each gene.

### The episignature of TTV enriches pathways involved in immune response

**Fig. 3:**
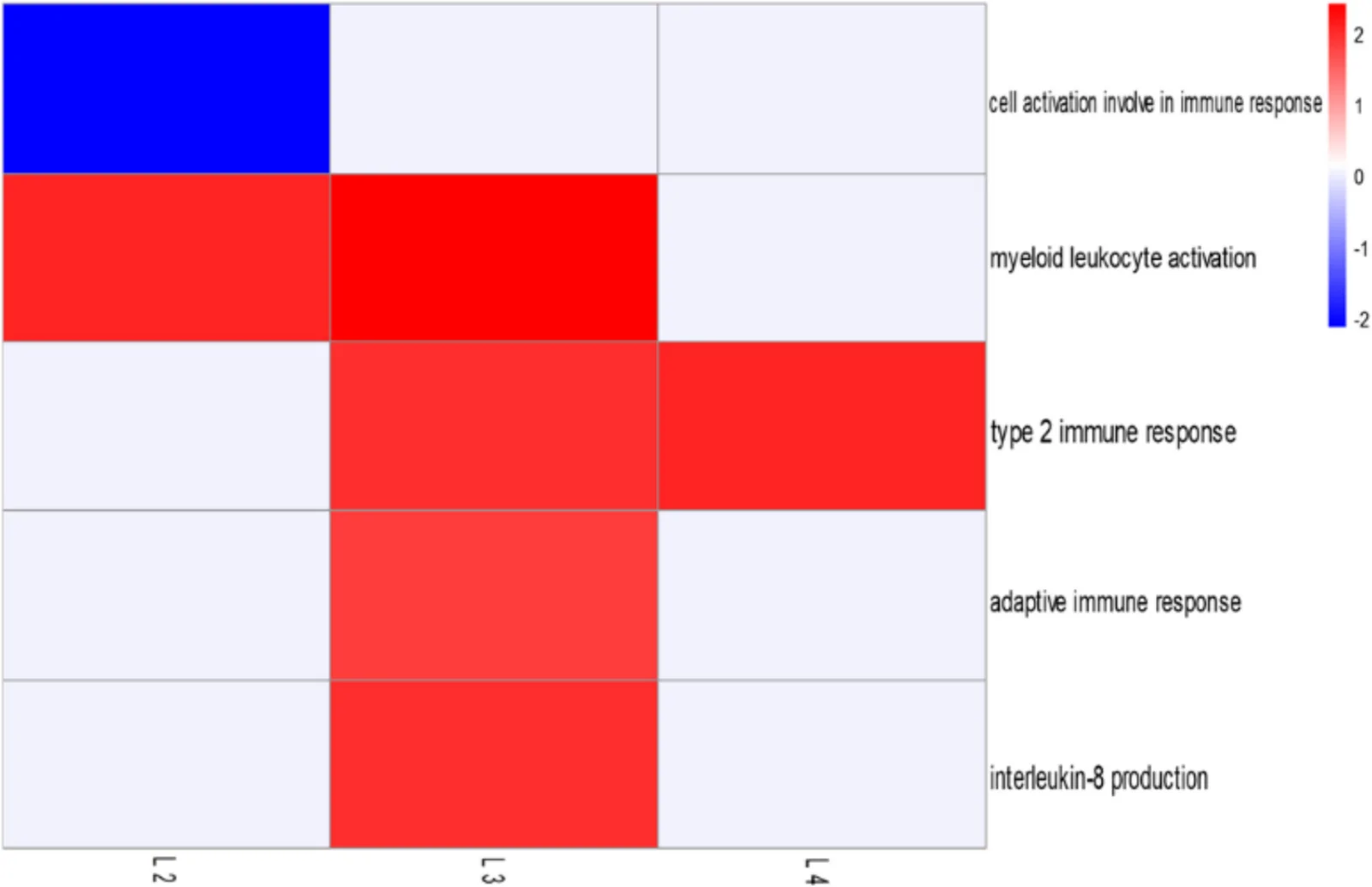
Heatmap of significantly enriched immune-related pathways (FDR < 0.25) based on the input gene lists. Pathways are coloured according to their normalized enrichment score (NES): blue for NES < 0 and red for NES > 0

**Fig. 4:**
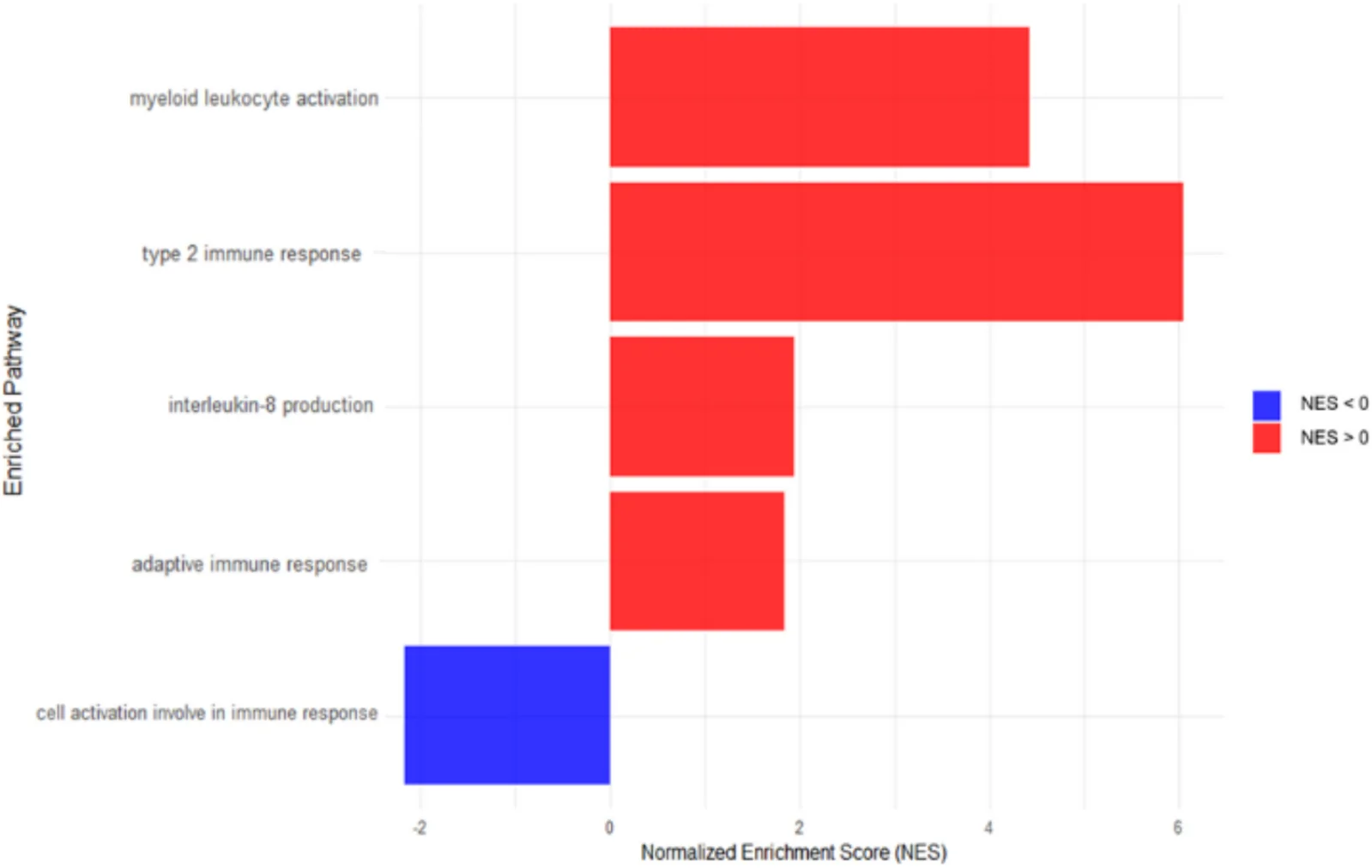
Bar plot of NES values for significantly enriched immune system pathways. Bars are coloured based on the sign of NES: blue for NES < 0 and red for NES > 0

GSEA were conducted on the WebGestAlt website and with the ClusterProfiler R package pipeline, using Gene Ontology – Biological Processes (noRedundant) as a reference for the functional database. The characteristics of the four input lists used in the analysis are summarized in Table S5. Complete lists, ranked according to each scoring method, are provided in Table S6. The complete data frames of clusterProfiler results, for each ranking score are reported in Table S7. As expected, L1 showed poor sensitivity for this approach, as it included the first 408 probes, corresponding to the first 378 most correlated genes and could not significantly enrich any gene set, regardless of the score used for ranking. Results from the lists from L2 to L4 revealed several pathways, including those related to the immune system. Both methods yielded comparable results when comparing the same ranking scores. The heatmap (Fig. 3) shows the enriched immune pathways that recur across multiple lists. In particular, the -log_10_(adj. *p*-value), logFC and logFC x -log_10_(adj. *p*-value) scores indicated a broad range of general biological processes such as “*cellular metabolic process*” (GO:0044237), “*regulation of cellular process”* (GO:0050794)*, “cellular response to chemical stimulus”* (GO:0070887), suggesting a systemic effect of TTV on the host organism. Nevertheless, all ranking scores were able to enrich some immune-related pathways, particularly for lists sorted by logFC: *“myeloid leukocyte activation”* (GO:0002274), *“type 2 immune response”* (GO:0042092), “*adaptive immune response*” (GO:0002250) and “*interleukin-8 production*” (GO:0032637). Notably, “*myeloid leukocyte activation*” was significantly enriched in L3 ranked by logFC (*p*-value < 2.2E-16 and an FDR = 0.03), but also in L2 ranked by logFC with a lower correlation; the ontology “*type 2 immune response*” appears with the strongest correlation among the results of L4 ranked by logFC (*p*-value < 0.01 and FDR = 0.09) but it is also present in the L3 ranked by logFC and in L4 ranked by logFC x -log_10_(adj. *p*-value) with a *p*-value < 0.001 and *p*-value < 2.2E-16, respectively, and an FDR = 0.2 for both analyses. Significantly enriched immune terms of WebGestAlt analyses are shown in Fig. 4.

**Fig. 5:**
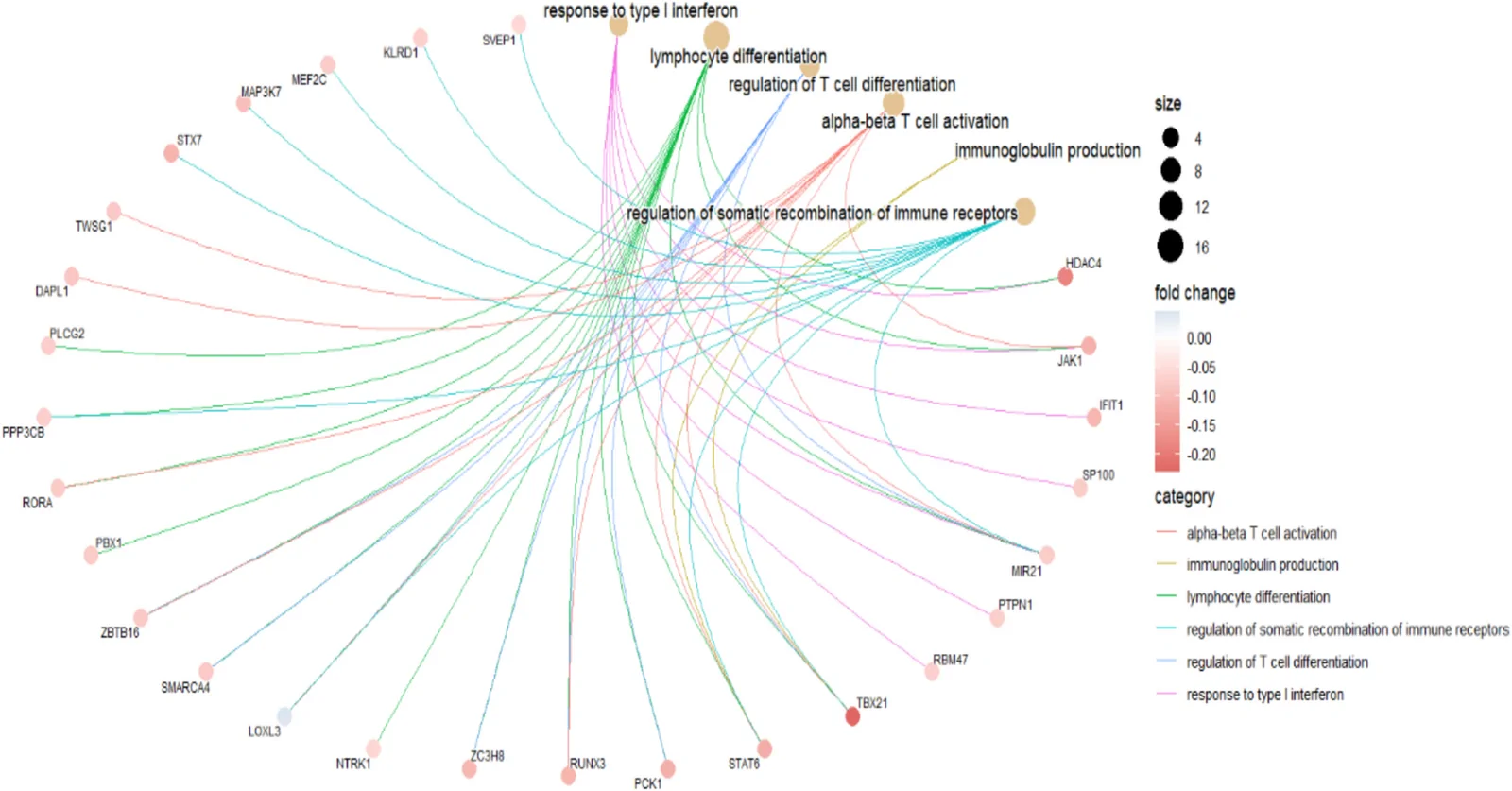
Gene-concept network of immune pathways enriched by hypomethylated DMPs. Gene nodes are coloured according with logFC; size of pathways nodes is proportional to the set size and edges colours depends on the enriched category. For clarity, the term GO:0002822 was labelled as “regulation of somatic recombination of immune receptors”

Concerning the hypomethylated positions (3623 genes), we found again a bunch of general pathways related to regulatory processes such as “*cell–cell signaling*” (GO:0007267) or “*signal transduction*” (GO:0007165), with an adj. *p*-value < 0.004 for the former and an adj. *p*-value < 0.001 for the latter. Those gene sets were mostly represented in ClusterProfiler results, but were also present with a higher FDR in WebGestAlt. Among immune-related pathways enriched in hypomethylated gene lists, we mention *“response to type I interferon”* (GO:0034340, adj. *p*-value < 0.03), *“lymphocyte differentiation”* (GO:0030098, adj. p-value < 0.004), “*regulation of T cell differentiation*” (GO:0045580, adj. *p*-value < 0.02), *“alpha–beta T cell activation”* (GO:0046631, adj. p-value < 0.02), and *“regulation of adaptive immune response based on somatic recombination of immune receptors built from immunoglobulin superfamily domains”* (GO:0002822, adj. *p*-value < 0.04). Figure 5 displays the relationships between the mentioned enriched GO terms and associated genes.

### The episignature of TTV enriches specific metabolic pathways

Further investigations on GSEA results identified additional significantly enriched gene sets not specifically related to the immune system. Firstly, we found in many outputs the term “*uronic acid metabolic process*” (GO:0006063). This pathway was strongly associated according to WebGestAlt algorithm with lists ranked by -log_10_(adj. *p*-value) with both *p*-value and FDR < 2.2E-16 and with the hypomethylated probes (*p*-value < 2.2E-16, FDR = 0.06). The analysis performed with ClusterProfiler showed a wider range of significantly enriched gene sets related to the glucoronidation process. Apart from “*uronic acid metabolic process*”, ClusterProfiler pointed out the involvement of additional ontologies such as “*glucoronate metabolic process*” (GO:0019585, adj. *p*-value < 0.04), “*flavonoid glucoronidation*” (GO:0052696, adj. *p*-value < 0.002) and the more generic “*cellular glucoronidation*” (GO:0052695, adj. *p*-value < 0.04) both for the hypomethylated and the -log_10_(adj. *p*-value) ranked list, respectively.

Secondly, we found “*protein localization to cell junction*” (GO:1,902,414) among significant results (*p*-value < 2.2e-16, FDR = 0.046974) for the -log_10_(adj. *p*-value) score. Similarly, the enrichment results of the hypermethylated positions were strongly converging into the “*cell–cell adhesion *via* plasma membrane adhesion molecules*” (GO:0098742) pathway. Interestingly, WebGestAlt presented this term at the top of the enrichment results for hypermethylated probes (*p*-value < 2.2E-16 and FDR = 0.15), and ClusterProfiler found only one significant association corresponding to “*homophilic cell adhesion *via* plasma membrane adhesion molecules*” (GO:0007156) with an adj. *p*-value < 0.002. Altogether, these findings support an involvement of cellular adhesion reprogramming in a context of cell migration driven by signal transduction pathways.

### Cross-validation

To evaluate the episignature derived from the limma regression model, we searched the EWAS DataHub and GEO Datasets for an external cohort that adequately met our study criteria. However, no suitable older adult cohort with both methylome array data and TTV load measurement was available for an external validation. Consequently, we adopted an internal cross-validation strategy.

As described in the Materials and Methods section, three predictive models were constructed: (1) using only methylation data, (2) including methylation data with age and gender, and (3) including methylation data and all covariates used in the differential methylation analysis. We set alpha = 1 to enforce a stringent LASSO regression, ensuring highly selective models, and minimizing the risk of overfitting. A 9:1 training-to-test ratio was used, providing 259 training samples and 29 test samples. This split was chosen to maximize the training data for model fitting, avoiding data leakage. For each training phase, we selected lambda values in order to minimize the Mean-Squared Error (MSE). For the training of Model (1) the optimal lambda of 0.3181 yielded an MSE of 1.478 (SE = 0.2117). When age and sex were added in Model (2), the best value of lambda was 0.2774 and minimized the MSE to 1.323 (SE = 0.1296); an additional improvement was observed in Model (3) which achieved the lowest error at lambda = 0.2372, with an MSE of 1.294 (SE = 0.1749).

This approach selected 69 predictors in Model (1), 32 in Model (2), and 26 in Model (3) out of 866,230 CpG sites (Table S8). The overlapping sites between the top 50 sites of the episignature (chosen as reference) and the predictors of each model were 12 for Model (1) and 10 in both Models (2) and (3). Notably, the probe cg11589966, regulating the expression of the SGCE:PEG10 genomic locus, was selected as the strongest predictor in all models according to the predictive coefficient. Furthermore, among probes with high predictive coefficients, there were cg05228610 and cg01447503, located in the LPIN1 and ZNF638 gene regions, highlighting the putative association between the regulation of these genes and TTV load.

## Discussion

Nearly 30 years after its discovery, scientific research is still engaged in understanding TTV interaction with the host immune system. Current studies are focused on elucidating the immunological dynamics underlying TTV persistence, its ability to regulate immune status, its interdependence with the host’s immune competence and the molecular mechanisms by which it evades immune surveillance. In this study, we examined DNA methylation variations associated with TTV load in a cohort of hospitalized older adult patients (age range: 62–102 years). We observed that patients with higher TTV levels were significantly older, reflecting age-related changes in viral replication, as previously reported [6].

We identified the methylation markers most strongly correlated with TTV load, thus supporting the that TTV viremia might induce some biological responses potentially modulating the epigenetic dysregulation at these loci Functional annotation of the differentially methylated positions (DMPs) revealed the involvement of a variety of biological processes, ranging from general cellular functions to immune-specific pathways, suggesting that TTV viremia may exert a broad impact on host cellular biochemistry. The differential methylation analysis was conducted considering possible confounders and the cellular heterogeneity of samples and revealed six strongly correlated (adj. p-value < 0.05) probes. The most compelling evidence to emerge points to the involvement of the top-ranking genes in immune regulation, as supported by current literature. We found probe cg09501109, located in the HOXB13 promoter. This gene is involved in prostate cancer onset, where it influences histone deacetylation levels of lipogenic effectors [39]. Originally characterized for its role in anterior–posterior axis differentiation during embryonic development, HOXB13 has recently garnered attention for its critical role in immunity and prognosis in rectal and prostate cancers [38, 40]. A 2022 study on pancreatic adenocarcinoma identified MROH9 among necroptosis-related genes associated with oncogenic processes, suggesting its role in patient prognosis [33]. A pilot study conducted in 2024 on COPD and lung cancer demonstrated that the circular RNA form of this gene, hsa-MROH9_0001, correlates with immune cell infiltration [34]. Additionally, in giant cell arteritis, positional gene mapping has implicated MROH9 within loci associated with immune and coagulation pathways, supporting its role in inflammatory and vascular processes [35]. ZNF638 is a member of the Zinc Finger Proteins (ZFPs), a large protein family typically involved in the regulation of cell proliferation, invasion, inflammation and apoptosis in several cancer models [45]. Notably, low levels of ZNF638 are considered a biomarker for clinical response to immune checkpoint inhibition by activation of innate anti-viral immune response [37]. In our results, ZNF638 showed significant hypomethylation at the promoter region (probe cg01447503, adj. *p* < 0.024). Increased ZNF638 expression plays a key role in the antiviral immune response and may reflect host compensatory mechanisms to counter viral presence. The SGCE:PEG10 genomic locus includes the paternally expressed gene 10 (PEG10), an oncogenic factor that is upregulated in diffuse large B cell lymphoma, where it promotes cell proliferation and resistance to apoptosis [41]. Further studies have characterized PEG10-derived peptides in complex with the placental heat shock protein gp96, demonstrating their ability to elicit T-cell responses against hepatocellular carcinoma, suggesting their potential as promising targets for immunotherapy [42]. Lastly, LPIN1 has been strongly associated with inflammatory processes and macrophage infiltration in sepsis [44].

Interestingly, most of the genes mentioned above are also involved in lipid metabolism. For instance, ZNF638 has been proposed as a novel regulator of adipogenesis, as it significantly increases the adipogenic process in vitro through physical interaction with CEBP-β and CEBP-δ**,** triggering adipocyte differentiation. Conversely, its knockdown downregulates the expression of adipocyte-specific genes [36]. Furthermore, the involvement of HOXB13 in cancer onset is closely connected with its methylation level, which modulates its ability to interact with histone deacetylase HDCA3 in prostate cells and thus suppress the expression of fatty acid synthase [39]. Studies on 3T3-L1 and HepG2 cell lines have demonstrated that LPIN1 overexpression leads to an increase in triglyceride synthesis and secretion. Consistently, LPIN1 knockdown in buffalo mammary epithelial cells resulted in a marked reduction in triglyceride content, suggesting a crucial role for LPIN1 in fatty acid metabolism [43]. A recent study explored the correlation between the high-density lipoproteins (HDL) receptor SR-BI (scavenger receptor class B type I) and gammaherpesvirus replication, highlighting the importance of lipid metabolism in regulating viral infections [46]. Notably, our team has already observed a negative association between TTV DNA load and total serum cholesterol (*p*-value < 0.004), suggesting a potential dysregulation of lipid metabolism during chronic TTV viremia [6]. In a previous study, triglyceride levels also emerged as significant predictors of both TTV load (*p*-value < 0.015) and TTV miRNA-t3b levels (*p*-value < 0.002) based on an automatic linear modelling approach [4]. The observed connection between TTV load and methylation status of lipid metabolism-related genes raises the possibility for the development of future biomarkers for monitoring metabolic-immune interactions in older adults.

Furthermore, hypomethylation patterns were found to be significantly associated with several immune-related processes as revealed through Gene Set Enrichment Analysis (GSEA), highlighting a connection between TTV and the host immune system. While hypomethylated markers were enriched in pathways related to inflammatory activation, hypermethylated loci were associated with pathways involved in cell–cell signalling and modulation of cell adhesion. These findings are consistent with the results of GSEA performed using alternative ranking scores, in which the enriched terms were referred to leukocyte activation and migration, respectively. In particular, our results support a possible link between TTV-load and production of the chemokine IL-8 through NF-κB. We found indeed that NFKBIZ and NFKBID genes (Table S6), which encode two inhibitors of NF-κB, were among the hypermethylated DMPs associated with TTV episignatures [47]. These results are coherent with the previous finding from our group which had already observed a slight correlation between TTV load and both plasma IL-8 levels and monocyte count [4]. Interestingly, the ORF2 protein of TTV has been previously associated with dysregulation of the NF-κB signalling pathway, which might influence in turn cytokine and chemokine production, immune receptor, and adhesion molecules expression [9].

Finally, significant enrichment scores were observed for terms related to uronic acid metabolism and generally to cellular glucoronidation processes. These pathways emerged in the analysis of hypomethylated loci and lists ranked by -log_10_(adj. *p*-value). The main contribution to these results relies on the differential methylation of the cg02789126 probe, located within a complex genomic locus comprising multiple genes encoding UDP Glucuronosyltransferase family enzymes (UGT1A10, UGT1A1, UGT1A6, UGT1A8, UGT1A4, UGT1A3, UGT1A9, UGT1A7 and UGT1A5). Nevertheless, the glucoronidation-related terms involve several additional genes among the DMPs for TTV, such as UGT2A1, UGT2A2 (probe cg09450342) and PRKCE (probe cg03958364). In this context, it is worth mentioning that uronic acid has already been described as an eliciting factor of immune activity [48, 49]. In 2017, Liu and colleagues tested the immunomodulatory activity of acetylated polysaccharide from *Cyclocarya paliurus*, mainly composed of arabinose, galactose, glucose, and galacturonic acid. Authors observed an increased production of pro-inflammatory cytokines, including TNF-α, IL-1β and IL-6 in murine macrophages [50]. Similar results were obtained by Zhang’s research team while testing isolated polysaccharide compounds from *Polygonum multiflorum* on splenocytes and macrophages. Cells treated with a higher content of uronic acid exhibit an increased production of IL-2 and TNF-α and phagocytosis rate [51].

It is tempting to speculate that endogenously produced uronic acid and its derivatives might contribute to the immune response activation, depending on epigenetic modulations and that all the correlations we present in this work might reflect the interplay between TTV and the host’s immune response. Nevertheless, we are aware of the limitations present in this study. First of all, our cohort consists exclusively of hospitalized older adult patients, therefore, a substantial portion of the methylome may be influenced by the intricate network of age- related acute and chronic diseases and the concomitant drug treatments each subject is undergoing. Consequently, the analysis of those patterns effectively reflecting TTV variance needed to account for several confounding factors. As described above, we adopted an FDR threshold of 0.25, aiming to perform an exploratory enrichment analysis. With this regard, we tested multiple models for M-values linear regression and multiple input lists for the enrichment analysis, evaluating the sensitivity of this approach and comparing results emerged with different ranking scores and with different pipelines. Finally, we reinforced the robustness of our findings by implementing a predictive model through cross-validation. However, the lack of comparable study populations in public datasets underscores the elusive nature of TTV and highlights the need for further studies. Indeed, this study is the first attempt to analyse TTV-related methylation patterns and to investigate their influence on the downstream metabolic pathways. Given the exploratory nature of the enrichment analysis, these findings should be interpreted as preliminary indications of biological processes potentially involved, rather than definitive evidence of mechanistic relationships.

In conclusion, this study provides the first epigenetic signature of TTV across the whole methylome and shows how DMPs with a higher correlation with TTV load are involved in immune response, in particular, IL-8 production via NF-κB signaling. It also demonstrates that TTV-driven immune activation is linked to cell migration and junction remodeling, indicating broader effects on host physiology, including potential dysregulation of lipogenesis and core cellular metabolism.

Supplementary information.

## Supplementary Information

Below is the link to the electronic supplementary material.

ESM 1 (DOCX 213 KB)

## Data Availability

The datasets analyzed in this study are available from the corresponding author upon reasonable request.
