# Supplementary material for: Methylation patterns associated with TTV load in geriatric hospitalized patients: an exploratory functional analysis
Source: GeroScience. 2025 Oct 22;48(4):5427–43. doi: 10.1007/s11357-025-01938-6 (PMC13575009; doi:10.1007/s11357-025-01938-6)
Supplement: Supplementary file 1 [file 11357_2025_1938_MOESM1_ESM.docx]

**Supplemental Figures**

**Figure S1**

**Fig. S1 Iterative Principal Components Analysis for methylation levels ranked by logFoldChange.**

(A) Euclidean distance of the centroids of the two clusters of patients (above and below median of logTTV) in the PC1 and PC2 space in function of the number of CpGs included in the PCA. (B) Variance explained by the first two components of the PCA in function of the number of CpGs included. (C) Area under ROC curve variation referred to the first two components predictivity towards the two groups of patients (above and below logTTV median) in function of the number of CpGs included in the PCA. (D) Scatter plot. Patients are coloured according to their viral load, above (blue) or below median (red). Centroids and their distance are shown as black crosses and a dotted line, respectively. Pearson correlation: PC1/logTTV: ρ = -0.71; PC2/logTTV: ρ = 0.26

**Figure S2**

**Fig. S2 Iterative Principal Components Analysis for methylation levels ranked by logFoldChange x -log10(adj. p-value).**

(A) Euclidean distance of the centroids of the two clusters of patients (above and below median of logTTV) in the PC1 and PC2 space in function of the number of CpGs included in the PCA. (B) Variance explained by the first two components of the PCA in function of the number of CpGs included. (C) Area under ROC curve variation referred to the first two components predictivity towards the two groups of patients (above and below logTTV median) in function of the number of CpGs included in the PCA. (D) Scatter plot. Patients are coloured according to their viral load, above (blue) or below median (red). Centroids and their distance are shown as black crosses and a dotted line, respectively. Pearson correlation: PC1/logTTV: ρ = -0.24; PC2/logTTV: ρ = -0.63

**The supplementary material (Tables S1–S8) can be accessed at the following link:**

<https://www.dropbox.com/scl/fi/yt09879818acm3nm39tby/supplementary.xlsx?rlkey=squjf9jcoyaq3lxcxhspd8745&st=m7kpr8ka&dl=0>
